# Intra-molecular origin of the spin-phonon coupling in slow-relaxing molecular magnets†
†Electronic supplementary information (ESI) available: Methods and theory details, unit-cell optimized geometry, computed vibrational frequencies and anisotropy tensor scan along normal modes. See DOI: 10.1039/c7sc02832f



**DOI:** 10.1039/c7sc02832f

**Published:** 2017-07-31

**Authors:** Alessandro Lunghi, Federico Totti, Stefano Sanvito, Roberta Sessoli

**Affiliations:** a School of Physics , CRANN and AMBER , Trinity College Dublin , Dublin 2 , Ireland . Email: lunghia@tcd.ie; b Universitá degli Studi di Firenze , Dipartimento di Chimica “Ugo Schiff” , Via della Lastruccia 3-13, 50019, Sesto Fiorentino , FI , Italy . Email: roberta.sessoli@unifi.it

## Abstract

We perform a systematic investigation of the spin-phonon coupling leading to spin relaxation in the prototypical mononuclear single molecule magnet [(tpa^Ph^)Fe]^–^. In particular we analyze in detail the nature of the most relevant vibrational modes giving rise to the relaxation. Our fully *ab initio* calculations, where the phonon modes are evaluated at the level of density functional theory and the spin-phonon coupling by mapping post-Hartree–Fock electronic structures onto an effective spin Hamiltonian, reveal that acoustic phonons are not active in the spin-phonon relaxation process of dilute SMMs crystals. Furthermore, we find that intra-molecular vibrational modes produce anisotropy tensor modulations orders of magnitude higher than those associated to rotations. In light of these results we are able to suggest new designing rules for spin-long-living SMMs which go beyond the tailoring of static molecular features but fully take into account dynamical features of the vibrational thermal bath evidencing those internal molecular distortions more relevant to the spin dynamics.

## Abstract 2

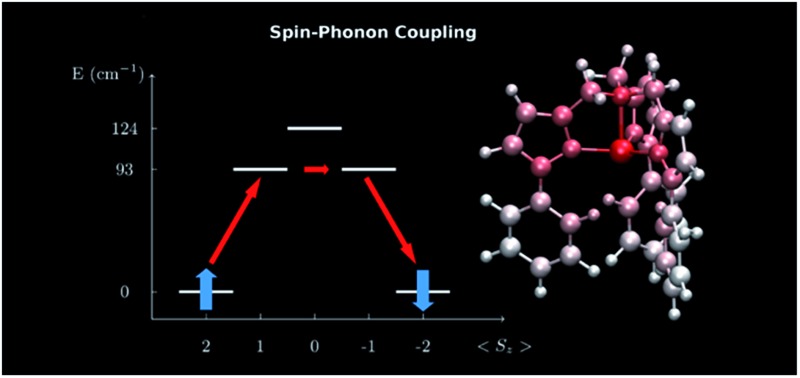
The design of slow relaxing magnetic molecules requires the optimization of internal molecular vibrations to reduce spin-phonon coupling.

## 1 Introduction

Single molecule magnets (SMMs) are open-shell metal complexes, which show a magnetic bi-stability thanks to their intrinsic magnetic anisotropy.^1^ The rich quantum mechanical behaviour displayed by this class of molecules has generated a strong interest and possible applications in fields such as high-density memory storage,^2^ spintronics^3^ and quantum computing^4^ have all been envisioned. One of their crucial characteristics is the slow magnetization relaxation, which has inspired a two-decades long study of spin dynamics with the final goal of extending the molecules' operating temperature up to the ambient one.

The simpler description of the temperature-activated spin relaxation in SMMs is in terms of an Arrhenius-like law, namely *τ* = *τ*_0_ exp (*U*_eff_/*k*_B_ *T*), where *τ* is the spin life-time, *τ*_0_ is the inverse attempt frequency setting the process time-scale and *k*_B_ is the Boltzmann constant.^5^ Here *U*_eff_ represents the analogous of the potential barrier of a diffusive process and it is generally referred to as the effective spin-flip energy barrier. This appears to be the central quantity when one wants to enhance the magnetization life-time, *τ*. In particular *U*_eff_ has been associated to the magnitude of the spin–orbit coupling inside the SMMs metal ions and this connection has been used as a design guideline to increase *U*_eff_.^6^ Although extensive molecular structure manipulations and ligands design have given us effective barriers as high as 1800 K with high temperature hysteresis,^7^ significant deviations from the Arrhenius law are observed on lowering the temperature. The latter have been mainly attributed to tunnelling effects^8,9^ but recently also spin-phonon interaction has been pointed to as responsible for these deviations. While optimization of magnetic anisotropy is nowadays based on sound design criteria no practical synthetic guidelines have yet emerged to control phonon effects. These facts clearly evidence our incomplete understanding of the spin-relaxation mechanism and the need of extending the current SMMs design strategies, so that molecules presenting long-living spin-states can be synthesized.

The magnetic bi-stability of SMMs is a non-equilibrium property. Ultimately the main source of relaxation for the spin at finite temperature is determined by the interaction with the environment vibrations, namely the spin-phonon coupling. Spin-phonon relaxation, also sometime called spin-lattice relaxation, is ubiquitous in chemistry and physics as it is generally responsible for relaxation in magnetic materials.^10,11^ Addressing the fundamental questions about its microscopic quantum origin is of paramount importance for modern material-science applications. For instance, it is crucial in quantum information processing, where spin-phonon relaxation is the ultimate origin of decoherence of solid-state electron spin qubits at room temperature.^12,13^

The current state of the art in the design of new and high-performing SMMs only concerns the tailoring of the static spin properties, namely reaching higher *U*_eff_ values. In such framework the environment and how this actually couples with the magnetic degrees of freedom is completely neglected. So far, a connection between the spin-phonon coupling intensity and the molecular crystal details has been stressed only at a phenomenological level, through the so-called Debye model.^5,10^ This essentially postulates that the acoustic phonons are solely responsible for the spin relaxation in molecular magnets. Despite its first formulation is more than fifty years old and the fact that a proof of its validity has never been provided, the Debye model remains the basis for the interpretation of every experiment to date. Over the last five decades, librational^14^ and optical modes^15^ have also been considered as possibly active in the spin relaxation process and have been phenomenologically included in the theory to overcome its limitations. However, until now the model has never been put to the scrutiny of a first principles theory, which can provide a rigorous demonstration of the real origin of spin-phonon relaxation in molecular magnets. Only lately some first few steps in this direction have been moved.^16–18^

Recently we have proposed a formulation of the spin-phonon equations of motion suitable for their computational implementation within a first principles framework.^19^ This is a full *ab initio* approach to the calculation of the spin-phonon relaxation in SMMs, which has enabled us to predict, for the first time, the spin relaxation time-scale of a mono-nuclear SMM. Our approach has thus provided important insights into the effect of the density of states and the phonons life-times on the temperature dependence of the Orbach and direct spin relaxation mechanisms.

Here we further exploit our computational formalism to establish a rigorous one-to-one relation between the spin-phonon coupling magnitude and the chemical nature of the vibrational spectrum. This is done for the prototypical SMM, [(tpa^Ph^)Fe]^–^,^20^ where the ligand H_3_tpa^Ph^ is tris((5-phenyl-1*H*-pyrrol-2-yl)methyl)amine. The present work, therefore, has the following goals: (i) to present the computational tools enabling the robust computation and analysis of the spin-phonon coupling coefficients for magnetic materials; (ii) to provide the first demonstration that intra-molecular vibrations offer the prominent contribution to spin-phonon interaction in dilute molecular compounds rather than rotational and translational motions as commonly assumed; (iii) to provide insights into the relation between the spin-phonon coupling magnitude and the structural features of molecular compounds; (iv) to provide new designing rules for the extension of the spin life-time in SMMs. These results represent a significant step forward into the comprehension of the spin-relaxation mechanism in molecular compounds and they provide new tools for the chemical control of these processes.

## 2 Computational methods

### 2.1 Inter- and intra-molecular vibrations decomposition

Our previously presented DFT calculation of the *Γ*-point normal modes of vibration of [(tpa^Ph^)Fe]^–^ (ref. 19) has been here used as the starting point for the new analysis of the spin-phonon coupling mechanism. This analysis has been performed through the decomposition of the normal modes of vibration into molecular local translational, local rotational and internal cartesian displacements. We have first selected the cartesian displacements of just one [(tpa^Ph^)Fe]^–^ molecule from each unit-cell normal modes and then we have decomposed them in the aforementioned contributions, following the procedure outlined by Neto *et al.* ^21^ (the mathematical details of this procedure have been summarized in the ESI†).

### 2.2 Spin-phonon coupling calculation and decomposition

The ORCA software^22^ has been employed to compute all the magnetic properties of the SMM. Calculations of the **D** anisotropy tensor have been performed at the CASSCF level with a def2-TZVP basis set for Fe and N, def2-SVP for C and H and a def2-TZVP/C auxiliary basis set for all the elements. This choice has been carefully tested and it correctly reproduces both the spin–orbit (SO) corrected spectrum and the **D** derivatives with respect to the molecular cartesian coordinates. These are calculated by employing the def2-TZVP basis set for all the atomic species. A (6,5) active space has been chosen as recommended in literature^23^ and the SO contribution has been included through quasi-degenerate perturbation theory. The calculation of the spin-phonon coupling coefficients has been performed following a **D** anisotropy tensor differentiation procedure, which is new and more robust with respect to that described in our previous report.^19^

As before, the CASSCF calculations have been carried over the structure of one [(tpa^Ph^)Fe]^–^ molecule as obtained by the DFT optimization of the crystal unit-cell. However, the anisotropy tensor has been differentiated with respect to the atomic Cartesian positions and not with respect to the unit-cell vibrations. This approach makes it possible to reduce the number of derivatives to be computed from 684 to 195. The large gain in computational overheads, that comes without any information loss, is due to the fact that the number of independent spin-phonon coupling coefficients scales with the number of atoms in the SMM molecule, while the number of vibrations scales with the number of atoms in the unit cell, which is in general larger. Because of this speed up in the computation of the spin-phonon coupling coefficients it has been possible to increase the number of points used to numerically evaluate a single derivative from two to ten, for a total number 1950 CASSCF calculations. Each element of **D** has been fitted with a second order polynomial expression against the size of the cartesian displacement. Only those with an error on the linear term smaller than 5% have been further considered, for a total number of 956 out of 1755 derivatives retained. Numerical derivatives with a higher error have been considered to be affected too much by numerical noise and therefore are considered as not well determined. The cartesian derivatives of **D** have been then transformed into four different sub-spaces: the sub-space of the unit-cell normal modes of vibrations, the sub-space of local molecular translations, the basis of local molecular rotations and the basis of intra-molecular displacements. These sub-spaces have been defined in the previous subsection and the details of the analysis are available in the ESI.†

### 2.3 **D** tensor norm definition

The traceless **D** tensor, appearing in the Hamiltonian 
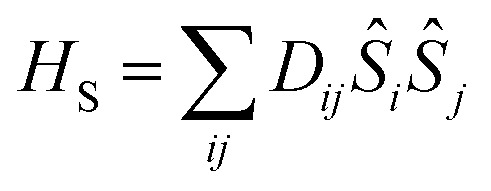
 for the spin, **S**, has five independent parameters. In order to make the analysis simpler it is convenient to construct a single invariant scalar quantity representing their average magnitude, as it is common practice for cartesian vectors. Let us start by noting that *H*_S_ can be written in an equivalent form as 
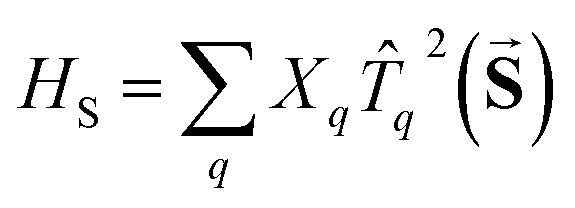
, where *T*_*q*_^2^(**S**) is a 2-rank spherical tensor. Since *H*_S_ is a 0-rank tensor the coefficients *X*_*q*_ must transform as *T*_*q*_^2^. It is then possible to exploit the composition theorem of spherical tensors^24^ to design the positive-defined scalar quantity1
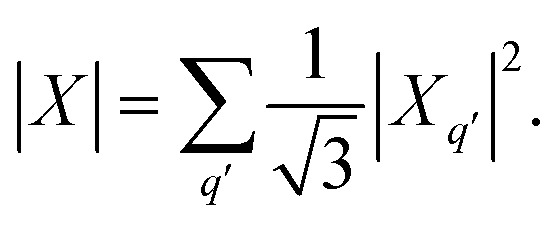



This serves as definition for the norm of **D**.

## 3 Results

### 3.1 Spin-phonon dynamics theory

The spin-phonon relaxation problem is stereotypical of an open quantum system. The first step to describe its dynamics consists in defining a global Hamiltonian including spin and phonon quasi-particles and their interaction. Let us assume that the magnetic properties of the SMM of interest can be described in a general way with a usual spin Hamiltonian,2
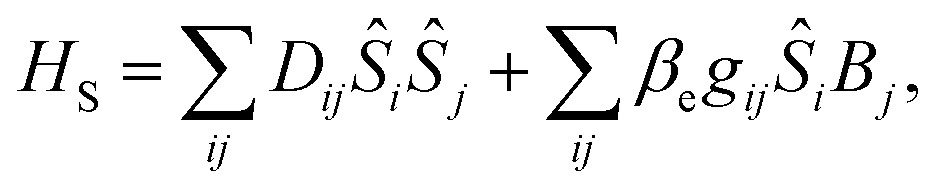
where *D*_*ij*_ and *g*_*ij*_ are the elements of the anisotropy and Landé tensors, respectively, *β*_e_ is the Bohr magneton, *B*_*i*_ are the components of the external magnetic field and *S*_*i*_ are the components of the molecular spin vector operator. The vibrational properties of a molecular crystal can be conveniently described with a Taylor expansion of the nuclei potential energy surface with respect to the normal modes of vibration, *q*_*α*_, with characteristic frequency, *ω*_*α*_,3
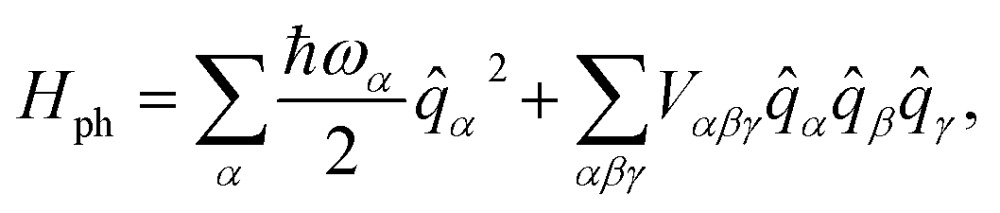
where *V*_*αβγ*_ are the anharmonic force constants and the summation on the index *α* is, in principle, extended to both vibrational bands and reciprocal-space *k*-points.

When the spin and the phonons are coupled it is possible to observe relaxation phenomena due to the energy exchange between the two subsystems. By assuming the dissipative dynamics of the spin to be much slower than that intrinsic of the vibrational thermal bath (Markov approximation), the equations of motion for the spin reduced density matrix, *ρ̂* ^S^, can be modeled according to the Redfield equation,^19,25^ which reads4
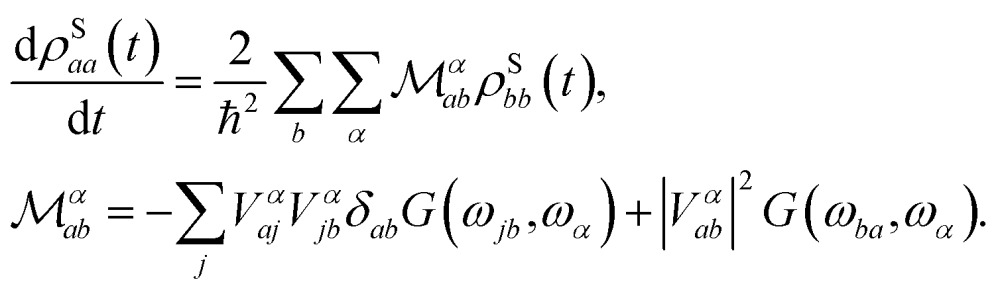



The use of this equation is justified by the fact that spin-relaxation for the system under study and similar SMMs occurs on the ms–μs time-scale, while vibrations posses a life-time in the ns–ps range.^19^ All the phonons contributions to the spin dynamics are described by the term, |*V* *α* *ab*|^2^ *G*(*ω*_*ba*_,*ω*_*α*_), that represents the kinetic rate of population transfer between the spin Hamiltonian's eigenstates |*S*_*a*_ and |*S*_*b*_, having populations *ρ*S*aa* and *ρ*S*bb*, respectively. In eqn (4) there are two fundamental quantities that drive the time evolution of the spin levels population: (i) the spin-phonon coupling matrix, *V* *α* *ab*, and (ii) the bath spectral function, *G*(*ω*_*ab*_, *ω*_*α*_), with *ω*_*ij*_ = (*E*_*i*_ – *E*_*j*_)/*ħ* and *E*_*i*_ being the spin Hamiltonian eigenvalue.

The interaction strength between spin and phonons, *V* *α* *ab*, can be described in the spin Hamiltonian formalism as5
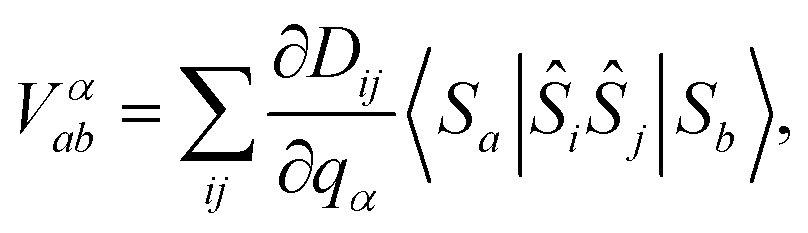
where the **D** tensor derivatives, *i.e.* the spin-phonon coupling coefficients, contain the magnitude of the coupling, while the bracket takes care of the selection rules given by *H*_S_ for the eigenstates |*S*_*a*_ and |*S*_*b*_. The spin-phonon coupling coefficients describe the interaction between the lattice vibrations and the local spin due to the presence of spin–orbit coupling, which ultimately is key for the energy exchange between nuclear and spin degrees of freedom.

Since here we wish to describe vibrations containing anharmonic terms, the bath spectral function, *G*(*ω*_*ij*_,*ω*_*α*_), assumes a Lorentzian shape6
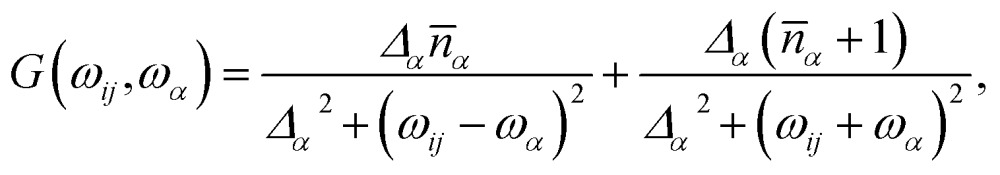
where 
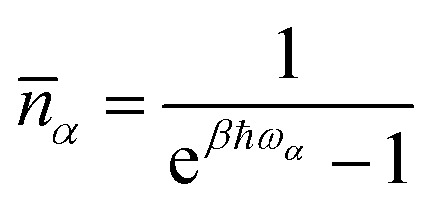
 is the mean phonon occupation number at temperature *T* and *β* = 1/*k*_B_ *T*. Finally, the quantity *Δ*_*α*_ is the *T* dependent life-time of the *α*-th phonon and its relevance has been discussed in our recent contribution.^19^ In a nutshell, its critical effect is to select, among the many phonons available, only those in near-resonance with the spin Hamiltonian eigenvalues, where the tolerance is set by the magnitude of *Δ*_*α*_. It must be stressed that the resulting relaxation rate is due to all these factors that act at the same time. However, when considering the *T* dependence of the spin relaxation, *n*(*T*) plays the important role of favouring more populated vibrational levels as source of spin-phonon relaxation. Therefore, in the commonly probed low-*T* regime,^20^ only the low-energy region of the phonon spectrum is expected to take part to the relaxation process. This effect is also even more emphasized by our modelling formalism due to the assumption that *Δ*_*α*_ becomes non-zero only at *T* values approaching *ħω*_*α*_/*k*_B_.

Spin-phonon coupling has also other effects beyond inducing the relaxation of the magnetization. For instance, as discussed in details by Escalera-Moreno *et al.* ^18^ for a highly coherent magnetic molecule containing a Cu^2+^ ion, non-zero homogeneous second-order derivatives of the spin-Hamiltonian with respect to phonons introduce a temperature dependent modulation of the spin energy levels splitting according to their coupling with the phonons. This effect has been previously investigated by us^19^ for the same system studied here and it is not discussed further.

This formalism has been recently used to successfully describe the first order spin-phonon relaxation of the single molecule magnet [(tpa^Ph^)Fe]^–^.^19^ The relevance of vibrational features like the phonon density of states (DOS) and the phonons life-time on the spin dynamics has been emphasized. However, in order to fully capture the effect of the phonons on the spin dynamics we also need to gain insights on the nature of the spin-phonon coupling coefficients. To make the spin relaxation time dependence on spin-phonon coupling more explicit let us consider the previously discussed simple case where one single strongly anharmonic phonon with frequency *ω* is resonant with one spin excited state with spin-phonon coupling coefficient *V*, leading to an Orbach relaxation.^19^ In this case the spin relaxation time *τ* is7
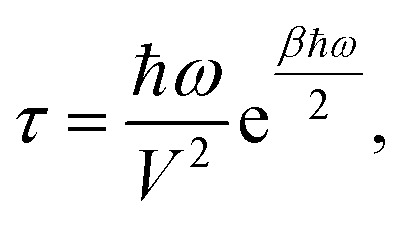
where the pre-exponential coefficient *τ*_0_ ∞ 1/*V* ^2^ and the spin-flip effective barrier *U*_eff_ = *ħω*/2. The magnitude of the spin-phonon coupling coefficients indeed sets the rate of both the Orbach and the direct relaxation mechanisms^10,19^ and the rationalization of their correlation with the molecular nature of the vibrations is of fundamental importance if one wishes to chemically engineer these quantities.

### 3.2 Results for [(tpa^Ph^)Fe]^–^

**Fig. 1:**
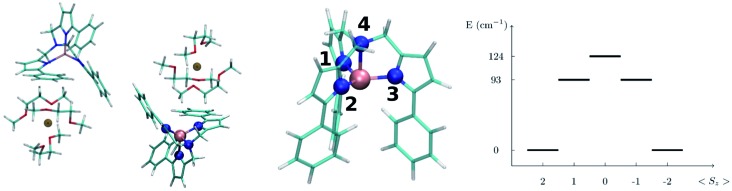
Left panel: the structure of the [Na(tpa^Ph^)Fe]^–^ 3 DME (DME = dimethoxyethane) unit cell. Central panel: zoom in on the molecular structure of [(tpa^Ph^)Fe]^–^. The color code is: Fe pink, N blue, C green, O red, Na brown and H white. The equatorial N atoms are marked by the labels 1 to 3 and the axial N atom by 4. Right panel: spin energy levels diagram obtained from the diagonalization of the computed spin Hamiltonian.

In this section we present the [(tpa^Ph^)Fe]^–^ SMM and show results for the computed spin-phonon coupling coefficients and how they originate from the molecular motion. The [(tpa^Ph^)Fe]^–^ crystal has a unit cell containing two molecular units and two Na^+^ cations coordinated by three dimethoxyethane molecules each,^20^ as showed in the left panel of Fig. 1. The symmetry of the [(tpa^Ph^)Fe]^–^ crystal is *P*1 and the two SMMs are related by inversion symmetry, so that all the results will be discussed by considering a single [(tpa^Ph^)Fe]^–^ molecule as representative of the entire crystal. The electronic structure of [(tpa^Ph^)Fe]^–^, calculated at the CASSCF level, shows that the five lowest-lying eigenstates corresponds to a *S* = 2 spin multiplet, whose degeneracy is lifted by spin–orbit coupling as schematically showed in the right panel of Fig. 1. This is in agreement with previous evidences.^20,23^ Such low-lying manifold of spin states can be described with the spin Hamiltonian *H*_S_ = *Dŝ*_*z*_^2^ + *E*(*ŝ*_*x*_^2^ – *ŝ*_*y*_^2^), equivalent to eqn (2) with the *z*-axis lying along the magnetization easy-axis. The axial anisotropy constant, *D* = –27.5 cm^–1^, and a vanishing *E*/*D* ratio grant to this molecule a slow relaxation rate of its magnetization in an external applied field. As previously discussed by others, the origin of this rather high axial anisotropy lies in the incomplete lifting of the orbital degeneracy in the [(tpa^Ph^)Fe]^–^ electronic structure.^23^ Indeed, the combination of the trigonal pyramidal coordination geometry (N_1–3_ are the equatorial ligands and N_4_ is the axial ligand in Fig. 1) and the high stiffness of the Fe^2+^ ligands cage had been specifically designed to hinder Jahn–Teller distortions and provide strong spin–orbit splitting. This is a general strategy used to build highly anisotropic molecular magnets^6,8,26–28^ and the results obtained here for [(tpa^Ph^)Fe]^–^ apply readily also to other systems in a rather general fashion.

The calculated spin-phonon coupling coefficients have been used to compute the effect of each unit cell *Γ*-point normal modes. It is important to remark that the choice to restrain this study to the *Γ*-point comes with no loss of information. Indeed, for each *k*-point a number of phonons equal in number to the unit-cell degrees of freedom is generated and so we are able to cover the full molecular configurational space already at the *Γ*-point. The full inclusion of the Brillouin zone integration in eqn (3) is however expected to be important to quantitatively estimate the spin life-time and it will be topic of future investigations.

**Fig. 2:**
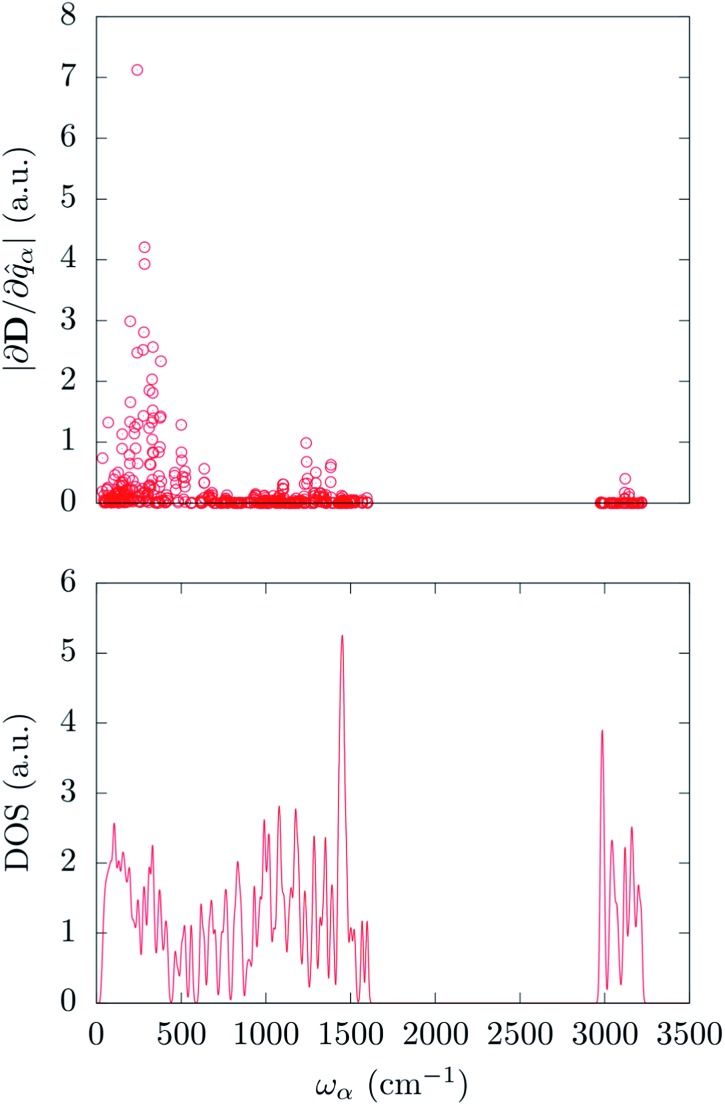
Top panel: calculated spin-phonon coupling coefficients projected onto the normal modes basis set and displayed as a function of the modes frequency. Bottom panel: DFT calculated density of states for the *Γ*-point normal modes of vibration.

**Fig. 3:**
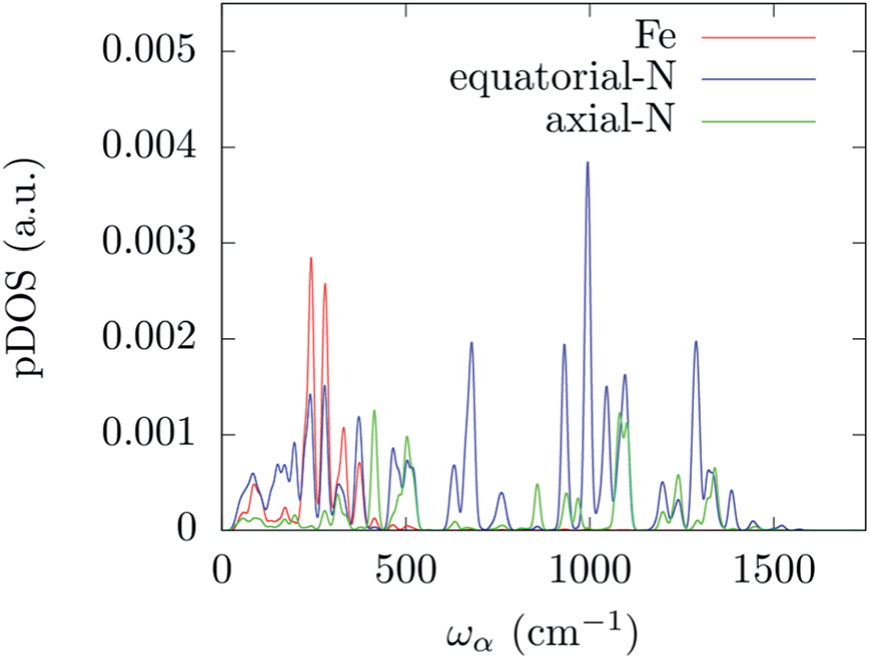
DFT calculated density of states for the *Γ*-point normal modes of vibration projected onto the metal and its first coordination shell atoms. The distribution has been evaluated by applying a Gaussian smearing of 10 cm^–1^.

In order to correlate the spin-phonon coupling coefficients with the normal modes it is convenient to use the spin-phonon coupling tensor norm |∂**D**/∂*q*_*α*_| as defined in the methods section. This invariant scalar quantity expresses the spin-phonon coupling coefficients magnitude for each vibration in a spherically averaged fashion. The computed |∂**D**/∂*q*_*α*_| are shown in Fig. 2 as a function of the frequency, *ω*_*α*_. The distribution of |∂**D**/∂*q*_*α*_| is rather non-uniform, suggesting that the nature of the normal modes is key when considering the rate of the spin relaxation processes. In order to address the origin of the non-uniform nature of |∂**D**/∂*q*_*α*_| we have analyzed the normal modes through the DOS and the projected DOS (pDOS) on the metallic core atoms. In Fig. 2 and 3 we present the total vibrational DOS at the *Γ*-point and those projected onto the first coordination shell of Fe^2+^, respectively. The DOS shows C–H stretching frequencies at around 3000–3250 cm^–1^ and a multitude of modes for frequencies smaller than 1500 cm^–1^. This is typical of coordination compounds. Notably, a large amplitude in the DOS is observed in the sub-500 cm^–1^ region. Such feature correlates with the large dimensions of the unit cell, which can sustain many low-energy delocalized normal modes. In discussing the pDOS we find that, as expected, the contributions of the Fe atom are localized in the low-energy region of the spectra due to its heavy mass. Although much lighter, also the N-ligands show a pronounced DOS amplitude in the low energy region since they are involved in the coordination bond with Fe.

**Fig. 4:**
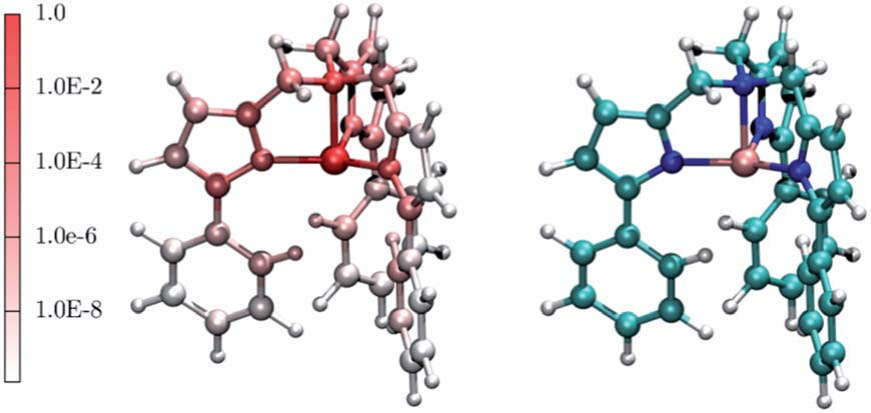
The red-to-white color scheme used for the molecule on the left-hand side panel represents the average atomic contribution to the spin-phonon coupling interaction averaged over the three spatial components and integrated over the entire energy spectrum. This value has been normalized to that of the Fe ion. As guide to the eyes, the right-hand side panel displays the [(tpa^Ph^)Fe]^–^ molecule. Colour code: Fe pink, N blue, C green, O red, Na brown and H white.

Interestingly, one can note correlation between the two main maxima of the |∂**D**/∂*q*_*α*_| distribution and the pDOS of Fe and N, centered at about 250 cm^–1^ and 1300 cm^–1^, respectively. The origin of this correlation is rather intuitive and originates from the fact that spin–orbit coupling, that is at the origin of the spin-phonon coupling, is a local quantity and it is maximized around the metal center (the heaviest of the atoms). This is further highlighted in Fig. 4 that shows the atomic projection of |∂**D**/∂*q*_*α*_| integrated over the entire phonon spectrum. As expected, the larger contribution to the spin-phonon coupling comes from the displacement of the Fe ion. It is interesting to note that although the contribution of the ligands is rather small when compared to that of Fe, as the different scales in Fig. 4 suggest, this does not vanish.

In order to understand which set of displacements is the most relevant for the relaxation process we need to decompose the normal modes of the molecular crystal into local molecular translations, local molecular rotations and intra-molecular vibrations. The same decomposition scheme has also been applied to the spin-phonon coupling coefficients in order to directly estimate the weights of the specific molecular distortions (see methods section).

**Fig. 5:**
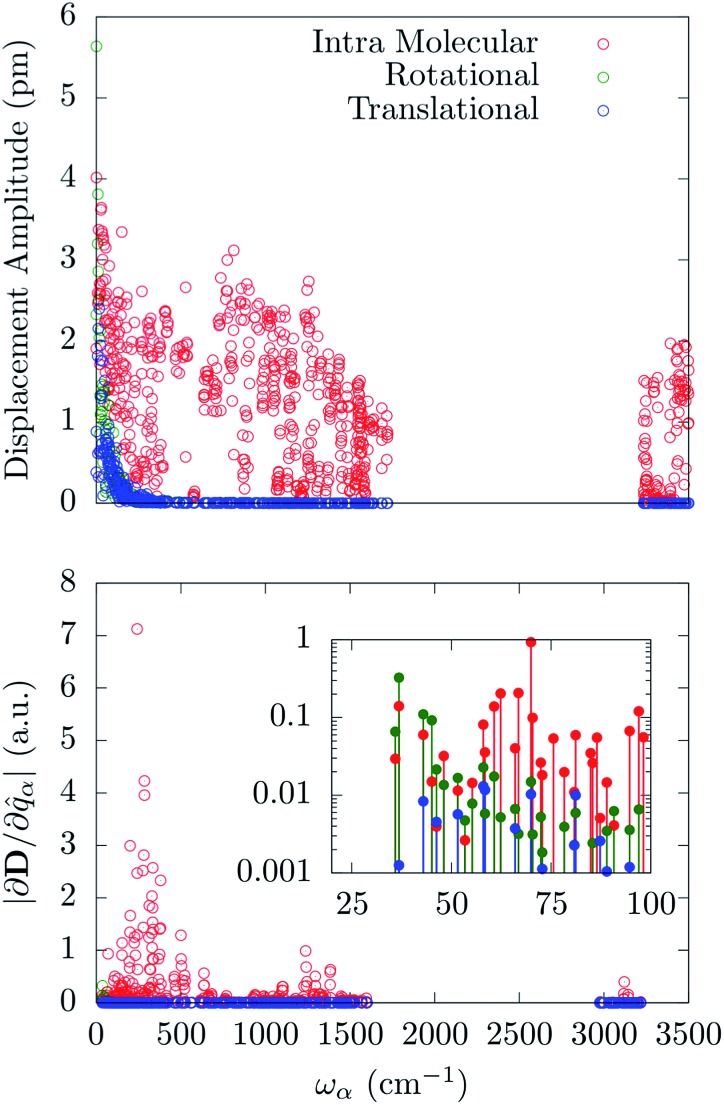
Upper panel: amplitude of the local translation, rotation and intra-molecular vibration as a function of the normal modes frequency. Bottom panel: magnitude of the spin-phonon coupling tensor norm decomposed onto the three different contributions as a function of the normal modes frequency.

Fig. 5 reports the amplitude of the specific molecular motions for each of the normal modes of the crystal, as a function of the frequency. All the local molecular rotations and translations are confined to the low-frequency region of the spectrum as generally expected for reticular vibrations. Importantly, there is not a clear separation between these molecular motions and the intra-molecular ones. The [(tpa^Ph^)Fe]^–^ molecule shows very low-energy-lying internal vibrations, which mix together with the external ones starting from the very first *Γ*-point normal mode. An attempt to further decompose the internal vibrations over stretching and bending distortions involving the first coordination shell shows that all these displacements are mixed inside the normal modes in a complex way. As such they cannot be easily grouped according to their frequency. Indeed, stretching and bending involving Fe and N atoms are present all over the sub-1500 cm^–1^ region with maxima qualitatively corresponding to the same maxima exhibited by the corresponding pDOS. The distribution of these quantities have been reported in the ESI.†

The spin-phonon coupling tensor norm, |∂**D**/∂*q*_*α*_|, has been used again to guide the analysis after the decomposition over the three possible types of molecular displacements. Our results are summarized in Fig. 5, where it is evident the prominent effect of intra-molecular displacements on the spin-phonon dynamics of [(tpa^Ph^)Fe]^–^. Remarkably, the contribution of the internal displacements on |∂**D**/∂*q*_*α*_| is almost indistinguishable from the total quantity displayed in Fig. 2 also in the sub 200 cm^–1^ energy window. This is where the local translations and rotations have non-zero amplitude. However, the inset of Fig. 5 shows that the very first few vibrations retain some contribution coming from rotational components of the normal modes. This can be attributed to the increase of weight of the external modes in this part of the spectrum, leading to an almost equal contribution of the internal and rotational motion in the first available normal modes. It is worth to point out that the translational motions never contribute to the spin-phonon coupling and that the very small fluctuations in Fig. 5 must be considered as numerical noise. This can be traced back to the space isotropy originating from the assumption of vanishing inter-molecular magnetic interactions. Indeed, a molecular translation cannot intrinsically produce any variation of the **D** tensor. This is not expected to hold when dipolar spin–spin interactions between different molecules/nuclei inside the crystal are considered. Spin dipolar interactions can also be added to our computational scheme in order to address phase decoherence processes and it will be topic of future investigations. The fact that only librational and optical modes contribute to spin-phonon coupling, at least in the framework considered here, further validates the *Γ*-point approximation used throughout this work. Indeed, librational and optical modes posses rather flat dispersions bands and the considerations about the spin-phonon coupling *vs.* frequency relations can be considered a good approximation of the full *k*-space spectrum situation.

**Fig. 6:**
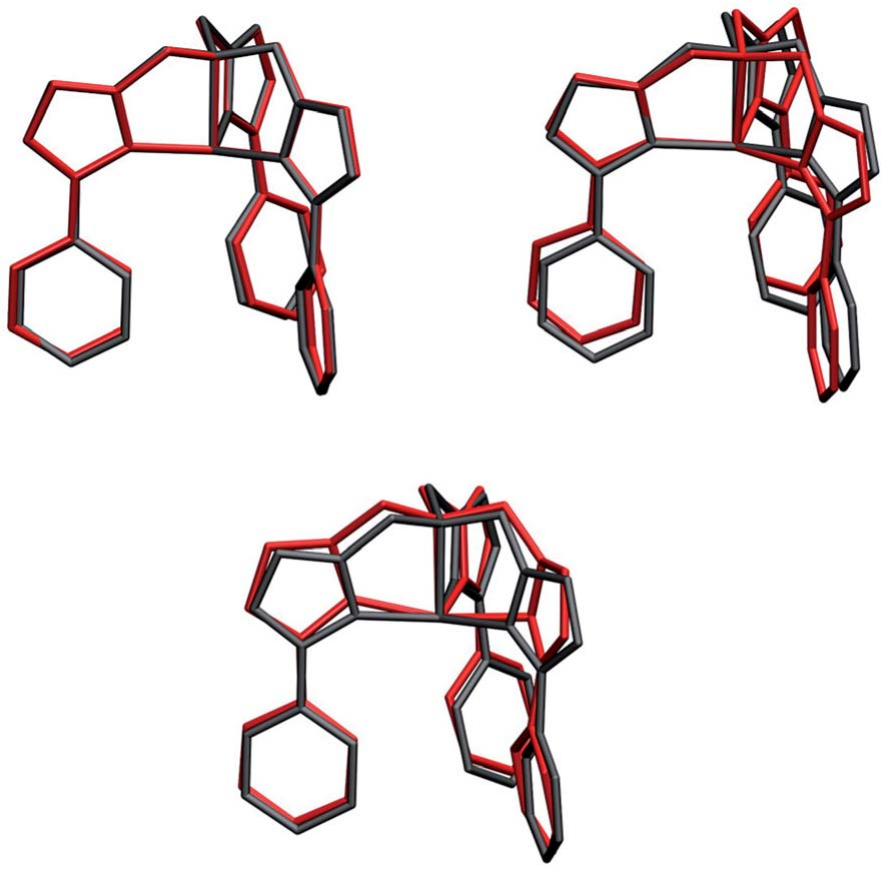
Molecular displacements originated by two different normal modes. Upper panel: intra-molecular (left) and rotational (right) displacements for the first available normal mode at ∼36 cm^–1^. Bottom panel: intra-molecular displacements for the spin coupled normal mode at 241.1 cm^–1^. Internal and rotational displacements correspond to 100 and 200 units of normal mode, respectively.

**Fig. 7:**
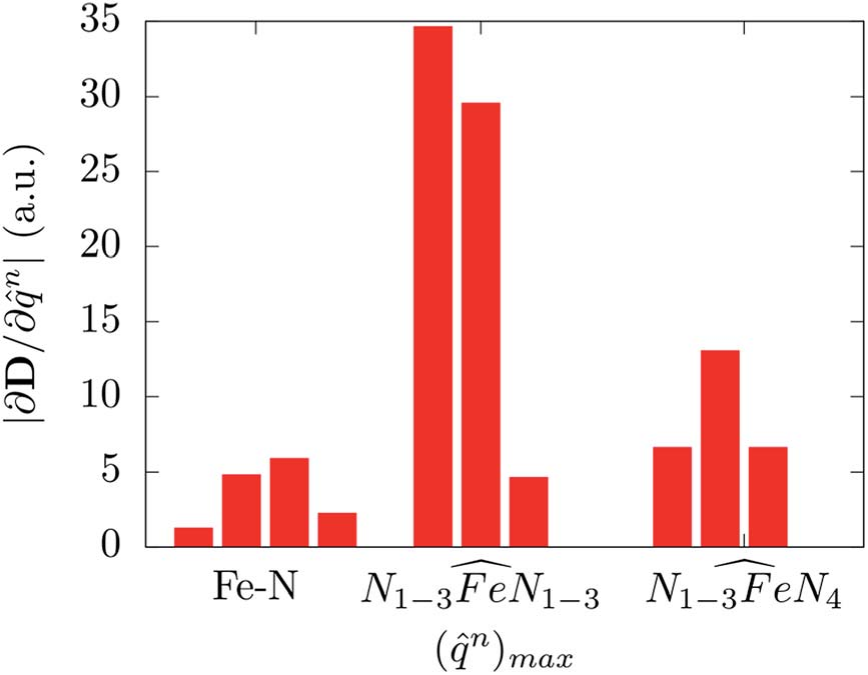
The magnitude of the spin-phonon coupling tensor norm is decomposed over ten internal coordinates *q* ^*n*^ corresponding to four Fe–N stretching, three in-plane and three out-of-plane 
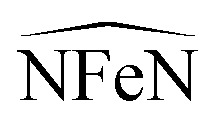
 bending modes involving or not the axial N_4_ atoms, respectively.

Molecular displacements associated to the modes at 35.9 cm^–1^ and at 241.1 cm^–1^, *i.e.* the first available normal mode and the one generating the highest spin-phonon coupling, have been reported in Fig. 6. Intra-molecular and rotational contributions to the first mode have been reported separately. The former show very small amplitude on the magnetic core and are quite delocalized on the ligands, giving the small spin-phonon coupling coefficient. The vibration at 241.1 cm^–1^ is dominated by intra-molecular distortions localized on the magnetic core, which produce a significant deviation of one 
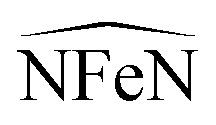
 bending angle involving two equatorial N atoms. This last result suggests the possibility to rationalize the spin-phonon coupling magnitude of [(tpa^Ph^)Fe]^–^ in terms of intra-molecular structural parameters, *q* ^*n*^, such as stretchings and bendings. Fig. 7 shows the result of this analysis where the spin-phonon coupling coefficients have been projected into internal parameters involving the first coordination shell and weighted for the maximum amplitude they assume along the whole phonon spectrum. Coherently to what observed in Fig. 6, the bending distortions involving the first coordination shell, and in particular the equatorial N ligands, are the most active source of spin-phonon coupling.

Finally, it must be recalled that, although the more interacting phonons are those around 250 cm^–1^, only the few low-lying vibrational states would be populated at the low temperatures explored by typical experimental investigations. In this scenario both rotations and internal vibrations will contribute to the relaxation as they are both present in the first modes. Moreover, due to the small Fe and N atoms' DOS in the low-energy region of the vibrational spectrum, the contribution to the spin-phonon coupling of more distant C atoms becomes not negligible.

## 4 Discussion

One of the central results of our simulations is the demonstration that the spin-phonon coupling coefficients due to the modulation of the molecular anisotropy are dependent on the nature of the vibration considered. Although not surprising, this result highlights the need to go beyond the conventional Debye model for spin relaxation in SMMs,^5,10,29^ where a Debye-like phonons dispersion with uniform spin-phonon coupling intensity simplifies the problem. Another shortcoming of the commonly used model is the assumption of a phonon spectrum dispersion consistent with acoustic phonons. In fact, we have here clearly demonstrated that intra-molecular vibrations, *i.e.* optical phonons, play a relevant role in the spin relaxation mechanism. In particular, it has been possible to show that specific internal vibrations are more strongly coupled with the spin center, namely those changing the 
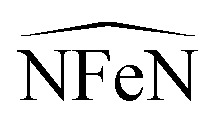
 angle. This last result opens up a critical discussion on the connection between molecular features and spin-phonon relaxation.

As briefly discussed before, the origin of the high anisotropy in transition metal molecular magnets has to be found in the fine tuning of the geometry of the first coordination shell. Transition metal molecular magnets, differently from lanthanide-based magnets, show a quenched angular momentum due to Jahn–Teller activity and for this reason high values of spin–orbit coupling can only be reached by a careful choice of ligands orientation so that the first excited state is as close as possible to the ground state.^23^ The Jahn–Teller distortions are then usually inhibited by designing the organic ligands to have a high stiffness. In this way the electronic degeneracy is only partly lifted and the high anisotropy is retained. Such recipe constitutes a designing rule of thumb for building slow-relaxing transition metal based SMMs, which has been proven to be remarkably efficient in the construction of highly anisotropic ground states. However, at the same time, the spin relaxation time-scales are still far from reaching the macroscopic values needed for practical applications. Our discussion sheds some light on this issue and provides new possible designing guidelines. The main idea is that the same structural features leading to high anisotropies do also enhance the spin-phonon coupling. Indeed, forcing the first coordination shell into a Jahn–Teller-unstable geometry has the consequence that any residual vibration coupled to the quasi-degenerate ground state, not fully hindered by ligands stiffness, is highly effective in changing the magnetic anisotropy and therefore in inducing a strong spin-phonon coupling. Concerning the specific case of [(tpa^Ph^)Fe]^–^, we find that the equatorial 
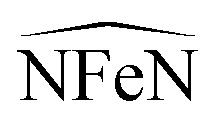
 angle modulations are the most coupled motions to the spin and indeed, as discussed by Atanasov *et al.*,^23^ these motions are also the main origin of Jahn–Teller activity inside this family of Fe^2+^ SMMs. In agreement with the analysis of Zadrozny *et al.*,^28^ we can suggest that this effect is also responsible for the observed strong reduction of *τ*_0_ in the Orbach's relaxation processes as the effective spin-flip energy barrier increases. Indeed, while the former is proportional to the spin-phonon coupling coefficients, the second is related to the energy of the phonon interacting with the spin excited states (see eqn (7)). Although the same arguments apply also to lanthanide-based magnet, the latter are expected to be less sensitive to these issues as the magnetic f-electrons are naturally less coupled to the ligands shell. This fact, together with their intrinsic high anisotropy, indeed made possible to observe much longer relaxation times for this class of magnets.^7^ In this regard, it is interesting to note how *S* = 1/2 systems,^30–32^ where zero-field-splitting is not possible at all, become competitive with highly anisotropic single-ion magnets, showing very long relaxation times at moderately high temperatures when a magnetic field is applied. In this emerging class of molecular magnets the design criteria is completely reversed, namely one should eliminate the origin of the spin-phonon coupling by using ions with quenched orbital angular momentum contribution.

According to our discussion, the anisotropy tensor and its derivatives seem to be unluckily correlated. However, it is worth reminding that, at least in principle, they are two physically unrelated quantities and that the correlation between the two is due, at least partially, to the specific design strategy used to build single-ion magnets. Nevertheless, the optimal control of all these different figures of merit leads to an extremely delicate designing process and a new generation of SMMs can only be proposed following a more thorough strategy. In this, static magnetism and spin-phonon dynamics features must be tuned at the same time. The computational strategy outlined in the present work can be applied to any SMMs and it can be helpful for the rational design of the spin-phonon coupling features, where common available experimental setups struggle to disentangle the many interactions involved.

**Fig. 8:**
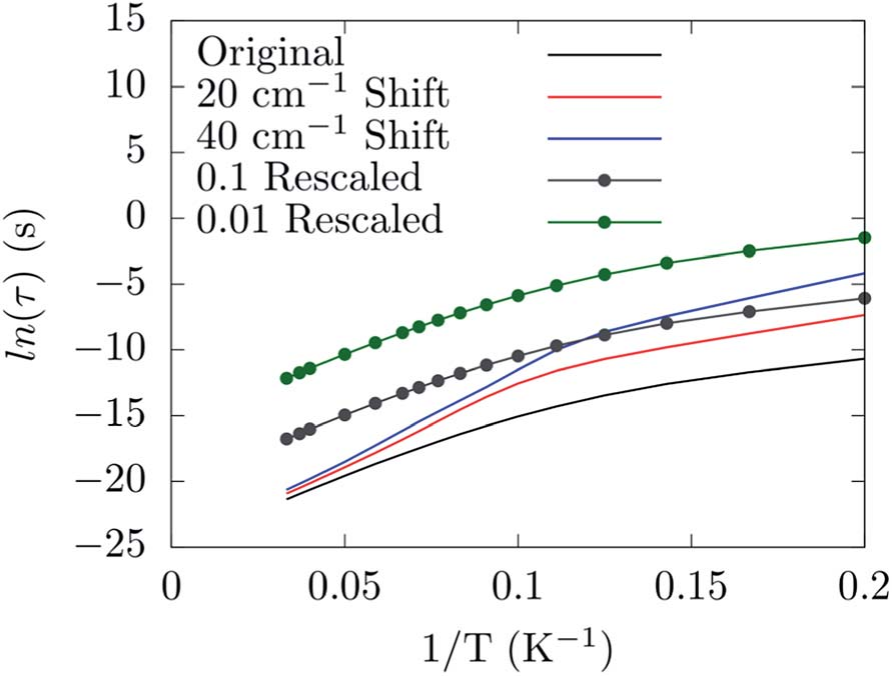
The dependence of the spin relaxation time on the spin-phonon coupling and phonons DOS tuning.

Already at this stage a few new guidelines for preparing a spin-phonon coupling-resistant SMM can be outlined. As shown by our analysis the most important vibrations are the low-energy-lying ones, since they are the most populated at the typical experimental temperatures. Moreover, the largest contribution to spin-phonon coupling comes from the first coordination shell. These facts indicate two possible strategies: (1) one should design the molecule so that the lowest-lying vibrational frequencies are shifted up in energy, hence the related phonon modes are only poorly occupied; (2) one should design the metal ligands so that the low-lying phonons have only a small contribution from the magnetic core region of the molecule in order to reduce the spin-phonon coupling magnitude of the more populated phonons. By solving the Redfield eqn (4) as previously discussed,^19^ it is possible to put on a semi-quantitative ground the effect of the proposed guidelines. Fig. 8 shows the predicted spin relaxation time as function of a separate rigid shift of the phonons' DOS and rescaling of all the spin-phonon coupling coefficients. The shift of the vibrational frequencies has a large effect in the low-T region where the relaxation is mediated by the direct mechanism^19^ and involves already a sparse vibrational density of states. The rescaling of the spin-phonon coupling coefficients has the effect, showed in Fig. 8, of uniformly reducing the magnetization relaxation rate. It is worth noticing that such a reduction is quite important due to the spin life-time dependence by the inverse square of the spin-phonon coefficients, as showed in eqn (7), suggesting that the latter proposed guideline, although not trivial to implement, might be quite successful. Finally we review a few possible routes for the implementation of the just discussed guidelines. In general we can expect that stiffer vibrational constants between the metal center and the ligands will shift up the vibrational frequencies involving these atoms. A similar effect would also be achieved by choosing lighter elements. At the same time, in order to decrease the delocalization of these modes in the low-energy vibrational spectra, a reduction of the molecular size would be beneficial, too. Indeed, if there are less degrees of freedom that can mix with the local coordination shell vibrations, the chance to observe largely delocalized modes will decrease. This last strategy would also help to increase the frequency of the local rotations as the molecular moments of inertia is reduced. The net effect would be of reducing even further the thermal population of the first available phonons in the spectrum. In agreement with this last statements, a correlation between molecular sizes and spin life-time can been observed for vanadyl based *S* = 1/2 molecular magnets.^33^ In that contribution the authors showed the link between the magnitude of the observed phonon bottle-neck and the sparsity of the vibrational density of states close to *ω* = 0 at the *Γ*-point. The effect discussed in that paper is, however, an extrinsic one, *i.e.* it depends on the crystal size and not on the single-molecule details of the spin-phonon coupling and it is more relevant for the direct relaxation rather than the Orbach one featured by SMMs. Nevertheless, a clear correlation between the spin life-time and the dimension of the crystals unit-cell was evinced. This correlation has been also discussed in a recent study,^34^ where THz infra-red spectroscopy had been combined with magnetometry measurements to reveal a link between the first *Γ*-point vibrational frequency and the field-dependent features of the spin-dynamics. Although less transparent, due to multiple contributions to the relaxation, a similar trend between spin-phonon coupling intensity and molecular sizes can also be speculatively expected to be key to explain the dynamics observed for homo-ligands members of the linear Fe^2+^ series studied by Zadrozny and Atanasov *et al.* ^16,28^ In this last example, although no significant variations of the first excited state energy were computed upon changing the ligands' organic scaffold, the spin dynamics was found to be accelerated upon increase of the molecular dimensions.

## 5 Conclusion

We have addressed the question of the nature of the spin-phonon coupling in mononuclear SMMs. The SMM [(tpa^Ph^)Fe]^–1^ has been used as a benchmark system for the *ab initio* calculation of its spin-phonon relaxation dynamics. For this SMM we have shown the relative importance of the external and internal displacement contributions to the spin Hamiltonian modulations. We have demonstrated that acoustic phonons are not active in the spin-phonon relaxation process of dilute SMMs crystals, and that intra-molecular vibrational modes produce anisotropy tensor modulations orders of magnitude higher than those associated to rotations. Nevertheless, the thermal population function selects only a few modes to be operative at low temperature. As these are the modes with the lowest frequency, they are made by a mixture of external and internal displacements. Therefore both rotations and internal vibrations are responsible for the relaxation in this range of temperature. This picture can dramatically change at high temperature, when only internal displacements are expected to be operative. In the light of these results we have remarked the importance of taking into account lattice dynamics features besides the spin ones in the design of new long-living-spin SMM. This gives us new designing rules that can help in fabricating more robust SMMs.

## Conflicts of interest

There are no conflicts to declare.
