# Supplementary material for: Intra-molecular origin of the spin-phonon coupling in slow-relaxing molecular magnets
Source: Chem Sci. 2017 Jul 31;8(9):6051–9. doi: 10.1039/c7sc02832f (PMC5625570; doi:10.1039/c7sc02832f)
Supplement: Supplementary file 4 [file SC-008-C7SC02832F-s004.pdf]

Electronic Supplementary Material (ESI) for Chemical Science.
This journal is © The Royal Society of Chemistry 2017

Intra-molecular origin of the spin-phonon coupling in slow-relaxing molecular magnets
Supporting Information

Alessandro Lunghi*[a,b], Federico Totti[b], Stefano Sanvito[a] and Roberta Sessoli*[b]

Phonons' cartesian displacements and their decomposition.

A molecular system made by N interacting particles can be described in the harmonic approximation assuming the potential energy surface U to be well described by its Taylor expansion around the $T = 0K$ equilibrium position. In this circumstance the potential energy U reads

$$U(\vec{\mathbf{X}}) = \frac{1}{2}\sum_{ij}^{N}\sum_{st}^{3}\frac{\partial^2 E_{el}}{\partial X_{is}\partial X_{jt}}\Delta X_{is}\Delta X_{jt}\,, \tag{1}$$

where E_{el} is the adiabatic electronic energy and $\Delta X_{is} = X_{is} - X_{is}^{\circ}$ are the $3N$ cartesian displacements with respect to the equilibrium X_{is}° configuration, where i spans the atom indexes and s=x,y,z. This system can still be mapped on a set of 3N decoupled 1D harmonic oscillators by introducing the normal mode of vibration. We first start defining mass-weighted cartesian coordinates $u_a = \sqrt{m_i}\Delta X_{is}$, where the single index a=3(i-1)+s runs over the 3N degrees of freedom. Diagonalizing the force-constant matrix of the energy second-order derivatives $\mathbf{H}$ it is possible to define normal mode of vibrations q_a and their frequencies ω_a:

$$\frac{1}{2}\sum_{ij}^{N}\sum_{st}^{3}\frac{\partial^2 E_{el}}{\partial X_{is}\partial X_{jt}}\Delta X_{is}\Delta X_{jt} = \frac{1}{2}\sum_{ab}^{3N}\frac{\partial^2 E_{el}}{\partial u_a\partial u_b}u_a u_b = \frac{1}{2}\sum_{ab}^{3N}H_{ab}u_a u_b = \frac{1}{2}\sum_{a}^{3N}\hbar\omega_a q_a^2 \tag{2}$$

where given $\mathbf{H}$ eigenvectors L_{ab} and eigenvalues $diag(H)_{aa}$

$$q_a = \sum_{i}^{N}\sum_{s}^{3}\bar{L}_{ab}\Delta X_{is} = \sqrt{\frac{\omega_a}{\hbar}}\sum_{i}^{N}\sum_{s}^{3}L_{ab}\sqrt{m_i}\Delta X_{is}\,, \quad \text{with} \quad b = 3(i-1)+s \tag{3}$$

$$\hbar\omega_a = \sqrt{diag(\mathbf{H})_{aa}}\,. \tag{4}$$

The inverse transformation that defines the cartesian displacement associated to a unit-less normal mode $\bar{q}_a$ amount of displacement is therefore

$$\Delta X_{is} = \bar{L}_{ab}\bar{q}_a = \sqrt{\frac{\hbar}{m_i\omega_a}}L_{ab}\bar{q}_a\,, \quad \text{with} \quad b = 3(i-1)+s\,. \tag{5}$$

Cartesian displacements so obtained can now be decomposed in three contributions: translational, rotational and internal displacements. To do that we followed the procedure outlined by Neto *et al.*[1], where Eckart-Sayvets conditions are imposed on molecular atomic displacements through a self-consistent numerical procedure. The first step of this procedure consists in the definition of translational (T^{α}) and

[a] *School of Physics, CRANN and AMBER, Trinity College Dublin, Dublin 2, Ireland; E-mail: lunghia@tcd.ie*
[b] *Universitá degli Studi di Firenze. Dipartimento di Chimica "Ugo Schiff", Via della Lastruccia 3-13, 50019, Sesto Fiorentino (FI); E-mail: roberta.sessoli@unifi.it*

rotational (θ^α) displacements. This new set of coordinates however makes the metric tensor different from the identity matrix and both covariant and controvariant coordinates must be employed. The Einstein convention on repeated indexes will be used in the rest of the section.

$$T^\alpha = \left(\frac{\partial T^\alpha}{\partial X_{is}}\right)_0 \Delta X_{is} = \frac{m_i}{M_{tot}} \Delta X_{is} = \Delta X_{Bs} \tag{6}$$

$$\theta^\alpha = \left(\frac{\partial \theta^\alpha}{\partial X_{is}}\right)_0 \Delta X_{is} = m_i (I_{\alpha\beta})_0^{-1} M^\alpha_{st} (X^\circ_{it} - X^\circ_{Bt}) \Delta X_{is} \,, \tag{7}$$

where $(I_{\alpha\beta})_0$ is inertia tensor calculated in the centre of mass cartesian reference system and M^α, with $\alpha = 1-3$, are infinitesimal rotation matrices around the cartesian axis α:

$$\mathbf{M}^1 = \begin{vmatrix} 0 & 0 & 0 \\ 0 & 0 & -1 \\ 0 & 1 & 0 \end{vmatrix}, \quad \mathbf{M}^2 = \begin{vmatrix} 0 & 0 & 1 \\ 0 & 0 & 0 \\ -1 & 0 & 0 \end{vmatrix}, \quad \mathbf{M}^3 = \begin{vmatrix} 0 & -1 & 0 \\ 1 & 0 & 0 \\ 0 & 0 & 0 \end{vmatrix}. \tag{8}$$

In this framework, atomic positions with respect to the centre of mass $x_{is} = X_{is} - X_{Bs}$ can be calculated from the initial ones $x^\circ_{is} = X^\circ_{is} - X^\circ_{Bs}$ by summing the pure internal contributions ΔX^{int}_{is} and rotating the resulting coordinates:

$$x_{is} = \Lambda_{st} (x^\circ_{it} + \Delta X^{int}_{it}) \tag{9}$$

$$\Lambda = \mathbf{E} + sin(\psi)\mathbf{M}^\xi + (1 - cos(\psi))\mathbf{M}^\xi \mathbf{M}^\xi \,, \tag{10}$$

where Λ express a general rotation of an angle ψ around a vector ξ.
The amount of rotation in terms of Λ and translations can be determined by imposing the Eckart-Sayvets conditions to the internal displacements, that read

$$\left(\frac{\partial q^\alpha}{\partial X_{is}}\right) \Delta X^{int}_{is} = 0 \,, \qquad \alpha = \text{traslational or rotational coordinate} \tag{11}$$

This set of equations impose the independence of internal and external degrees of freedom, requiring that the projection of one set of coordinates onto the other is null. Indeed the term in parenthesis is nothing but the Jacobian matrix that transforms the complete set of 3N coordinates into the 6 external coordinates. Eqs.7 can then be used to compute the translational and rotational contribution of the full ΔX_{is} displacements, then the internal ones can be computed by difference through Eq. 9:

$$\Delta X^{int}_{is} = \Lambda^{-1}_{st} x_{it} - x^\circ_{is} \,, \tag{12}$$

where Λ can be calculated employing Eq. 10 and the relations

$$\psi = \sqrt{\theta^\alpha \theta^\alpha} \,, \quad \mathbf{M}^\xi = \frac{\theta^\alpha}{\psi} \mathbf{M}^\alpha \tag{13}$$

This process should be cycled using the calculated ΔX^{int}_{is} as new input displacements in Eq. 11 until the equation get verified. This procedure has been used to compute the amplitude of local translation, local rotation and internal vibrations of a single $[(tpa^{Ph})Fe]^{-1}$ molecule inside its crystal, as showed in the main text. To do so, we inserted in Eq. 11 only the 3N cartesian displacements corresponding to this specific

molecule coming from normal modes, which instead describe the vibrational motion of all the primitive-cell atoms. These quantities differ from the usually defined acoustic, librational and optical modes of a lattice as the latter are defined as translations, rotation and internal vibrations of all the atoms inside the unit-cell, with no particular distinction among inter/intra molecular motions.
To summarize, one starts with the normal modes provided by the DFT calculation. Next, by applying the projector technique to the normal mode cartesian displacements ΔX_{is}^{tot} of Eq. 5, one ends up with three additional kind of cartesian displacements: ΔX_{is}^{int}, ΔX_{is}^{rot}, and ΔX_{is}^{trasl}. By separately plugging these cartesian displacements in Eq. 4 one can define the normal modes projection onto the translational, rotational and intra-molecular space

$$q_a^{int} = \sum_i^N \sum_s^3 \bar{L}_{ab} \Delta X_{is}^{int} = \sqrt{\frac{\omega_a}{\hbar}} \sum_i^N \sum_s^3 L_{ab} \sqrt{m_i} \Delta X_{is}^{int}\,, \quad \text{with} \quad b = 3(i-1)+s \tag{14}$$

$$q_a^{rot} = \sum_i^N \sum_s^3 \bar{L}_{ab} \Delta X_{is}^{rot} = \sqrt{\frac{\omega_a}{\hbar}} \sum_i^N \sum_s^3 L_{ab} \sqrt{m_i} \Delta X_{is}^{rot}\,, \quad \text{with} \quad b = 3(i-1)+s \tag{15}$$

$$q_a^{trasl} = \sum_i^N \sum_s^3 \bar{L}_{ab} \Delta X_{is}^{trasl} = \sqrt{\frac{\omega_a}{\hbar}} \sum_i^N \sum_s^3 L_{ab} \sqrt{m_i} \Delta X_{is}^{trasl}\,, \quad \text{with} \quad b = 3(i-1)+s\,. \tag{16}$$

For completeness we here also report in Fig. 1 the contributions of acoustic, librational and optic motions as function of frequency. As expected, acoustic motions are absent at any frequency as we are looking only at Γ-point vibrations.

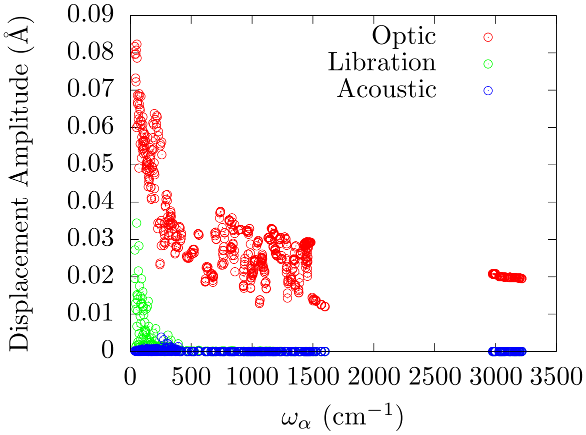

Fig. 1 Amplitude of acoustic, librational and optical motions for each normal mode and displayed as function of modes frequency.

The internal cartesian displacements have been further analysed in terms of internal coordinates, namely bending and stretching motions, involving the first coordination shell atoms. Figs.2 and 3 report the contribution of N-Fe stretching and NFeN bending motions to normal modes as function of frequency, respectively.

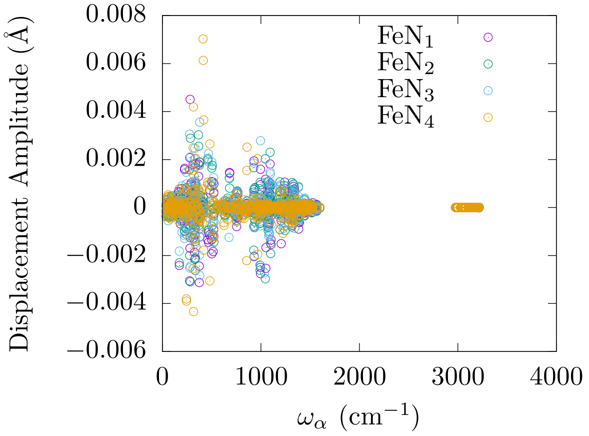

Fig. 2 Amplitude of stretching motions involving the central iron atoms and the first coordination shell N-ligands for each normal mode and displayed as function of modes frequency.

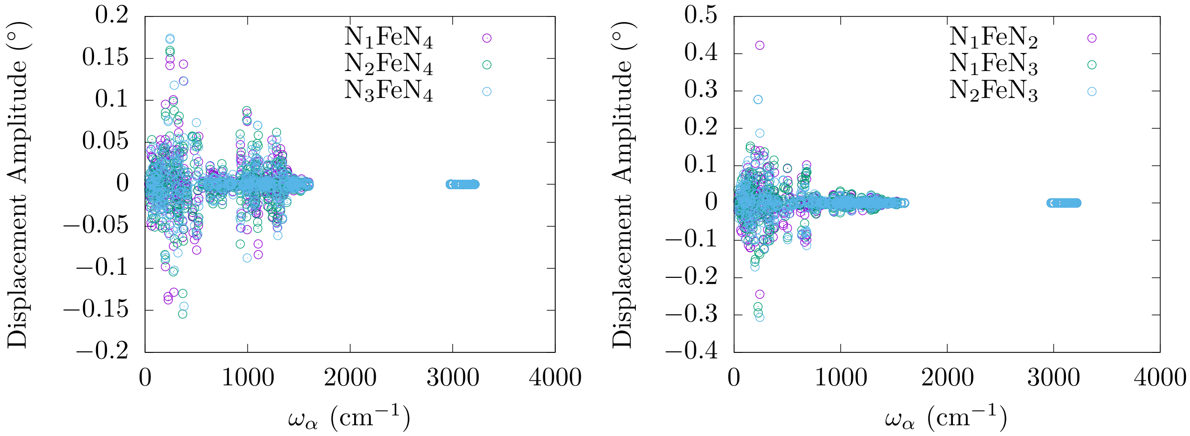

Fig. 3 Amplitude of bending motions involving the central iron atoms and the first coordination shell N-ligands for each normal mode and displayed as function of modes frequency. Two different plots have been used to distinguish those bending involving (left panel) or not (right panel) the axial N-ligand (N_4).

Spin-Phonon coupling coefficients calculation.

Spin-phonon coupling coefficients are defined as the first order derivatives of the spin Hamiltonian parameters with respect to the normal mode of vibrations $\partial D_{ij}/\partial q_\alpha$ [2]. The strategy we employed to compute them starts with the evaluation of the numerical **D** tensor derivatives with respect to the cartesian coordinates of $[(\mathrm{tpa}^{Ph})\mathrm{Fe}]^{-1}$. To do that we scanned one coordinate $X_{is} + \delta X_{is}$ at the time with steps δX_{is} equal to ± 0.0025, ± 0.002, ± 0.0015, ± 0.001 and ± 0.00075. The resulting points have then been interpolated with a second order polynomial expression in order to estimate the linear terms, that correspond to the $\partial D_{ij}/\partial X_{ks}$ coefficients. Here we would like to report a few examples to show the details of the method. Fig. 4 reports the scanning of the six independent **D** elements along the x component of the iron atom.

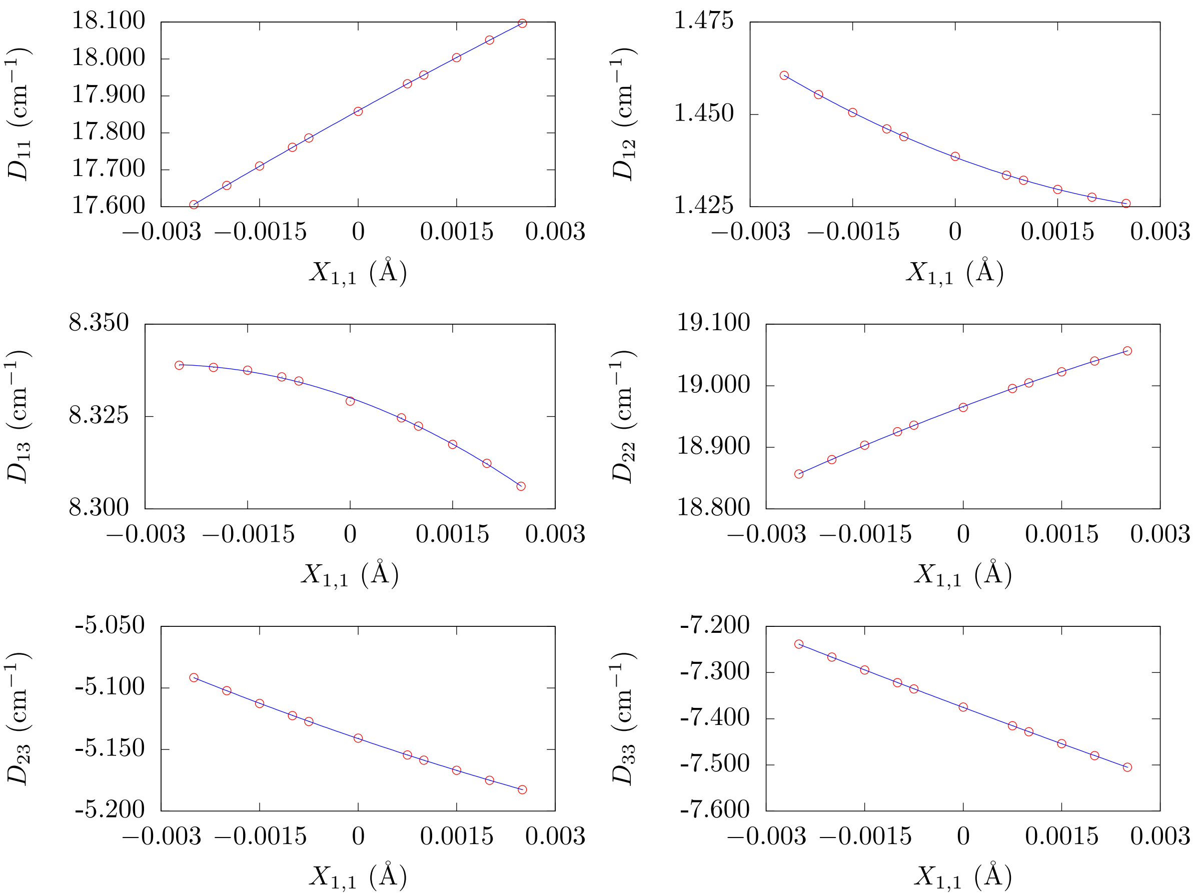

Fig. 4 Red circles correspond to the **D** anisotropy tensor independent elements D_{11}, D_{12}, D_{13}, D_{22}, D_{23} and D_{33} calculated along the x direction of the iron atom. The straight blue line corresponds to the second order polynomial fit function.

It is interesting to note how, in the case of iron coordinates derivatives, the second order component of the polynomial expression is fundamental to obtain a good fitting. This is not true however for all the other elements and for atoms outside the first coordination shell a linear expression is enough to obtain a good regression. The breaking down of a first order perturbation theory for large displacements of the first coordination shell clearly comes from the fact that spin-phonon coupling interaction is magnified in proximity of the iron d-shell electrons. For the same reason, increasing the distance between the displaced atom and iron, the value of spin-phonon coupling decreases and its evaluation become more affected by numerical noise. We decided to exclude D derivatives with an error on the linear term higher than 5%. As

an extreme example of excluded contribution, Fig. 5 reports the scanning of D elements along the y coordinate of a pyrrolide carbon not directly bounded to the nitro atom. Finally, the cartesian derivatives of

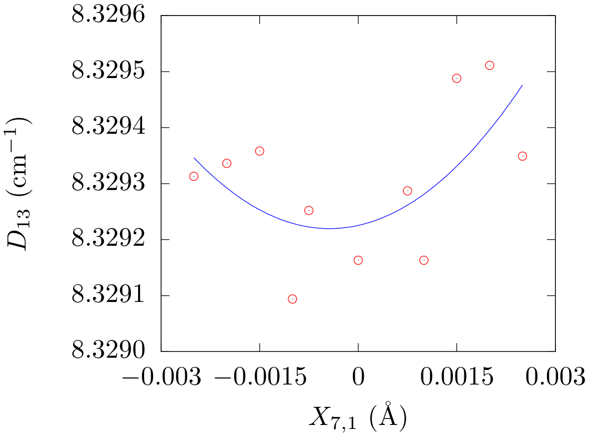

Fig. 5 Red circles correspond to D_{13} anisotropy tensor element calculated along the x direction of a carbon atom belonging to the pyrrolide group. The straight blue line corresponds to the second order polynomial fit function. Due to the evident large numerical noise we excluded this contribution from the analysis and considered it equal to zero.

the anisotropy tensor **D** have then been projected on the basis of normal modes by means of their cartesian coordinates definition in Eq. 5.

$$\frac{\partial D_{ij}}{\partial q_a} = \sum_i^N \sum_s^3 \frac{\partial X_{is}}{\partial q_a} \frac{\partial D_{ij}}{\partial X_{is}} = \sum_i^N \sum_s^3 \bar{L}_{ba} \frac{\partial D_{ij}}{\partial X_{is}} \quad \text{with} \quad b = 3(i-1)+s \tag{17}$$

This procedure for the computation of $\partial D_{ij}/\partial q_a$ has a double advantage. The first one regards the number of CASSCF calculations needed. Assuming the electronic structure of a single [(tpa^{Ph})Fe]$^-$ units to be independent from the other molecules in the crystal, the calculation of the **D** tensor derivatives can be done directly on a single molecule without periodic boundary conditions[2]. The number of independent displacements for a given molecule corresponds to its 3N degrees of freedom but the number of normal modes instead grow like the number of the primitive cell degrees of freedom, which is generally much larger than 3N. Moreover, Eq.17 can be used also to compute all the separate contributions to spin-phonon coupling coefficients by simply replacing $\bar{L}_{ba}$ with its own projection on specific displacements, calculated as explained in the previous section. This last step can be taken as many times as one wants as there are no additional computational costs beyond the $\partial D_{ij}/\partial X_{is}$ coefficients and phonons calculation already done. Fig. 6 reports the comparison between the spin-phonon coupling coefficients computed with the just outlined procedure and those obtained by the direct differentiation with respect to the unit-cell normal modes[2]. The two calculations show only minor differences, validating the consistency of both calculations.

One final remark regarding the possibility to project the spin-phonon coupling coefficients in the basis set of internal coordinates. To accomplish that the weighting coefficients of Eq. 17 must be chosen as $(\partial X_{is}/\partial q^n)$, where $q^n s$ represent controvariant internal coordinates such as stretchings bendings and torsions. The $q^n s$ are defined as function of cartesian coordinates and therefore the coefficients $(\partial X_{is}/\partial q^n)$ can be otbained by inversion of the Wilson's matrix $G_{n,is} = (\partial q^n/\partial X_{is})$. Although these coefficients are mathematically well defined this procedure hides a potential inconsistency. Indeed, due to the non-orthogonality of internal coordinates, this coefficients depends on the definition of the basis itself. For instance the computed derivative of a bending angle coordinate with respect to cartesian coordinates will

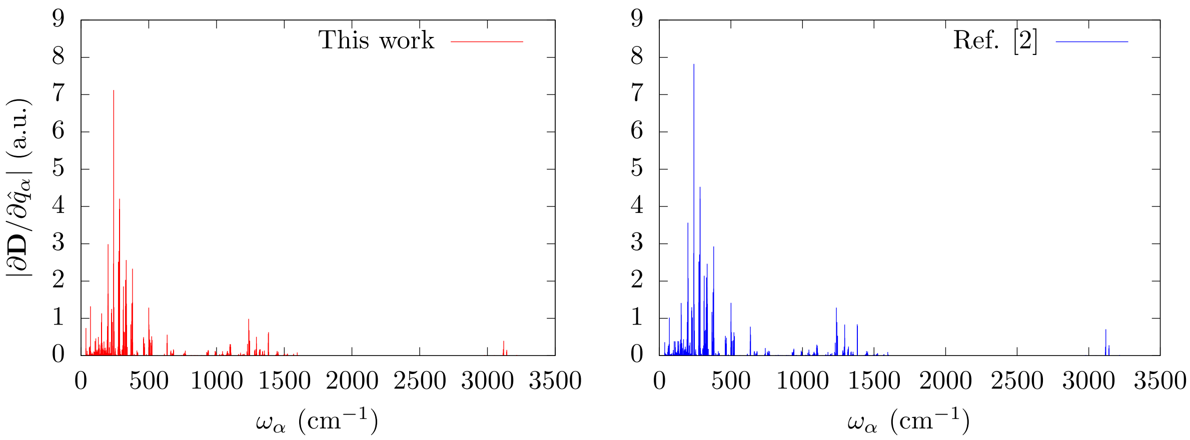

Fig. 6 Comparison between the spin-phonon coupling coefficients calculated by differentiation of cartesian coordinates (left panel) and by differentiation with respect to the normal modes (right panel).

be different depending on the set of 3N-6 internal parameters used to define the intra-molecular basis set. A possible solution to this problem regards the factorization of the metric tensor of the internal coordinates in order to define a new set of internal orthogonal parameter[3]. However, doing so the chemically intuitive picture in terms of stretchings and bending would be lost as one would be scanning the **D** tensor along a non-trivial combination of them. Consequently, we decided to keep working we the usual internal molecular coordinates and proceed as follows. Since we are interested in the Iron first coordination shell we selected three atoms at the time and compute the $(\partial X_{is}/\partial q^n)$ coefficients for the two stretchings and one bending. Doing so we obtain one $\partial D_{ij}/\partial q_n$ coefficients for each bending and three coefficients for each stretching, which have been avaraged among them. Using the smallest possible number of internal vectors at the time is then possible to reduce the uncerteinity on the computed coefficients due to the non-orthogonality issue. It must be stressed that the order of magnitude of the $\partial D_{ij}/\partial q_n$ coefficients is conserved within different basis sets. However, the correctness of this approach should be found in the coherence of the data presented in the main text.

References

1 N. Neto and L. Bellucci, *Chem. Phys.*, 2006, **328**, 259–268.
2 A. Lunghi, F. Totti, R. Sessoli and S. Sanvito, *Nat. Commun.*, 2017, **8**, 1–7.
3 N. Neto, *J. Mol. Struct.*, 2001, **563-564**, 135–139.
